# Supplementary material for: Non defect-stabilized thermally stable single-atom catalyst
Source: Nat Commun. 2019 Jan 16;10:234. doi: 10.1038/s41467-018-08136-3 (PMC6335577; doi:10.1038/s41467-018-08136-3)
Supplement: Supplementary file 1 — Supplementary Information [file 41467_2018_8136_MOESM1_ESM.pdf]

Non defect-stabilized thermally stable single-atom catalyst

Lang et al.

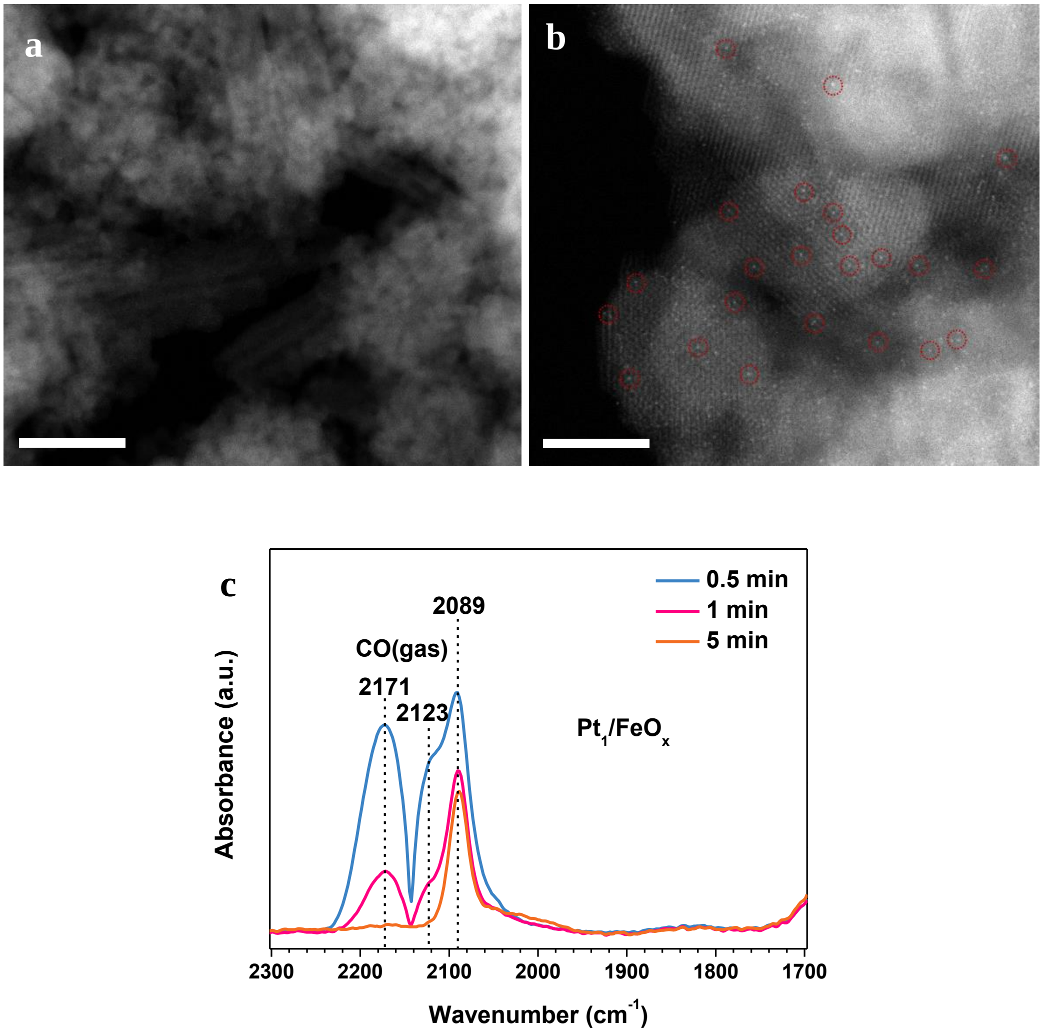

Supplementary Figure 1. Characterization of Pt_1/FeO_x.

(a) Dark-field STEM shows no evidence for Pt nanoparticles over the FeO_x support, 20 nm scale bar. (b) HR-STEM reveals isolated Pt atoms (circled in red), 5 nm scale bar. (c) DRIFT spectra of Pt_1/FeO_x were recorded after in situ heating at 200 °C under flowing He for 0.5 h, cooling to room temperature, saturation with CO (from a 1 vol% CO/He stream for 10 min), and subsequent flushing with He for 1-5 min to purge gas-phase CO (2,171 and 2,123 cm^{-1} band) from the environmental cell. The residual band at 2,089 cm^{-1} is assigned to linearly adsorbed CO; since this band position was independent of CO coverage it is attributed to molecules coordinated to isolated Pt atoms.

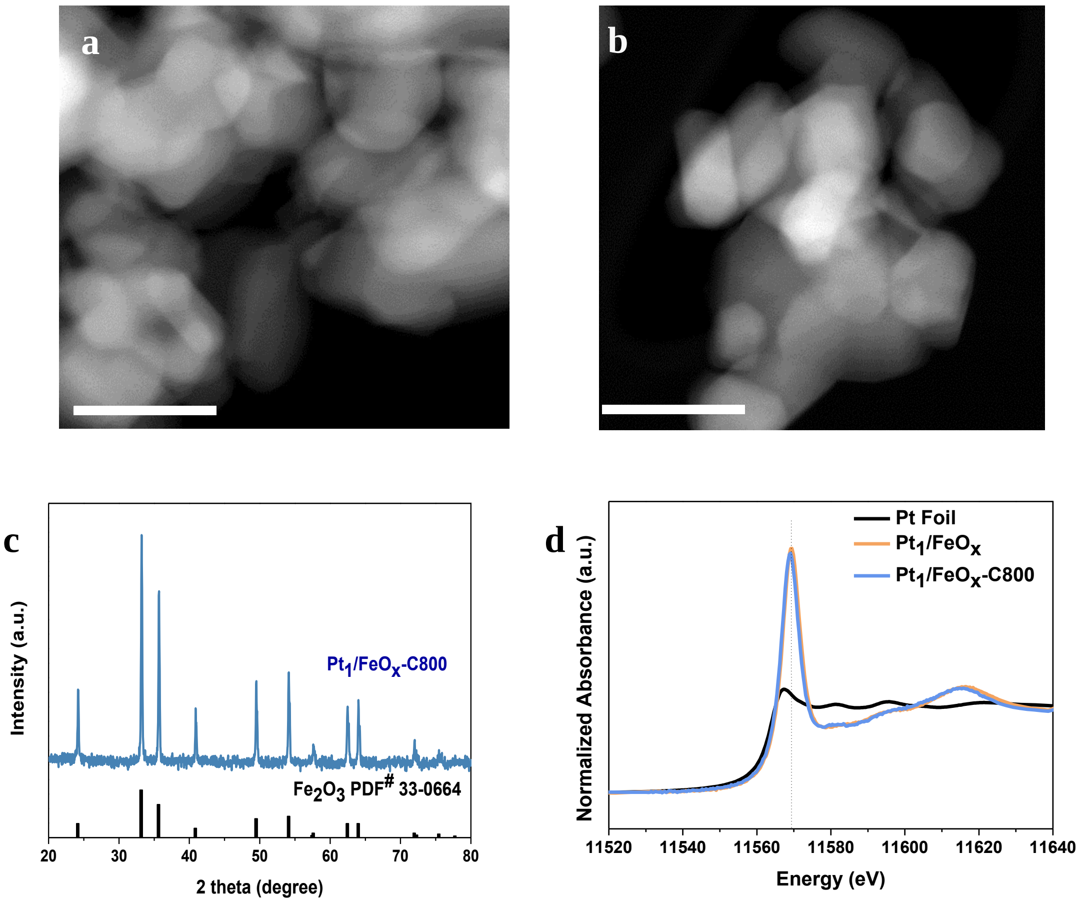

Supplementary Figure 2. Characterization of Pt_1/FeO_x-C800.

(a-b) No Pt particles were observed for Pt_1/FeO_x by HR-STEM following calcination at 800 °C for 5 h under flowing air, demonstrating that Pt remained highly dispersed as a low (mono-) nuclear species. Scale bars: 100 nm. (c) Powder XRD of Pt_1/FeO_x-C800 only showed reflections characteristic of α-Fe_2O_3, confirming the absence of >2 nm Pt nanoparticles. (d) Pt L_{III} XANES spectra of Pt_1/FeO_x before and after calcination, alongside a Pt foil reference, evidencing that the overwhelming majority of Pt species were in a high (likely +4) oxidation state.

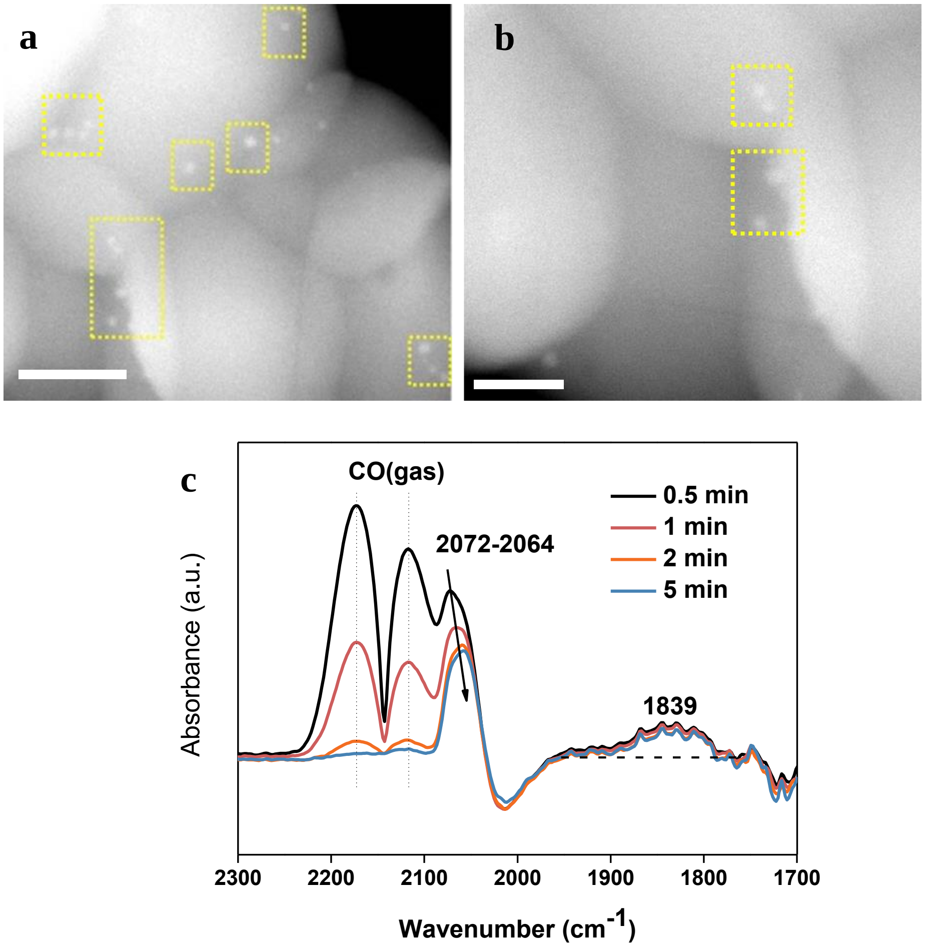

Supplementary Figure 3. Characterization of 0.3Pt/Fe_2O_3-NP.

(a-b) HR-STEM highlighting the presence of Pt NPs (yellow squares) over the Fe_2O_3 with a mean diameter of approximately 2 nm. 20 nm scale bar for panel a, and 10 nm scale bar for panel b. (c) DRIFT spectra of the 0.3Pt/Fe_2O_3-NP sample were recorded after in situ heating at 200 $^{\circ}$C under flowing He for 0.5 h, cooling to room temperature, saturation with CO (from a 1 vol% CO/He stream for 10 min), and subsequent flushing with He for 1-5 min to purge gas-phase CO (2,171 and 2,123 cm^{-1} band) from the environmental cell. The residual band at peak at 2,072 cm^{-1} is assigned to CO adsorbed on Pt NPs, which shifts to 2,064 cm^{-1} with decreasing CO coverage. The presence of Pt NPs is also indicated by the presence of a (weak) band at 1839 cm^{-1} characteristic of a bridge-bound Pt_2-CO species.

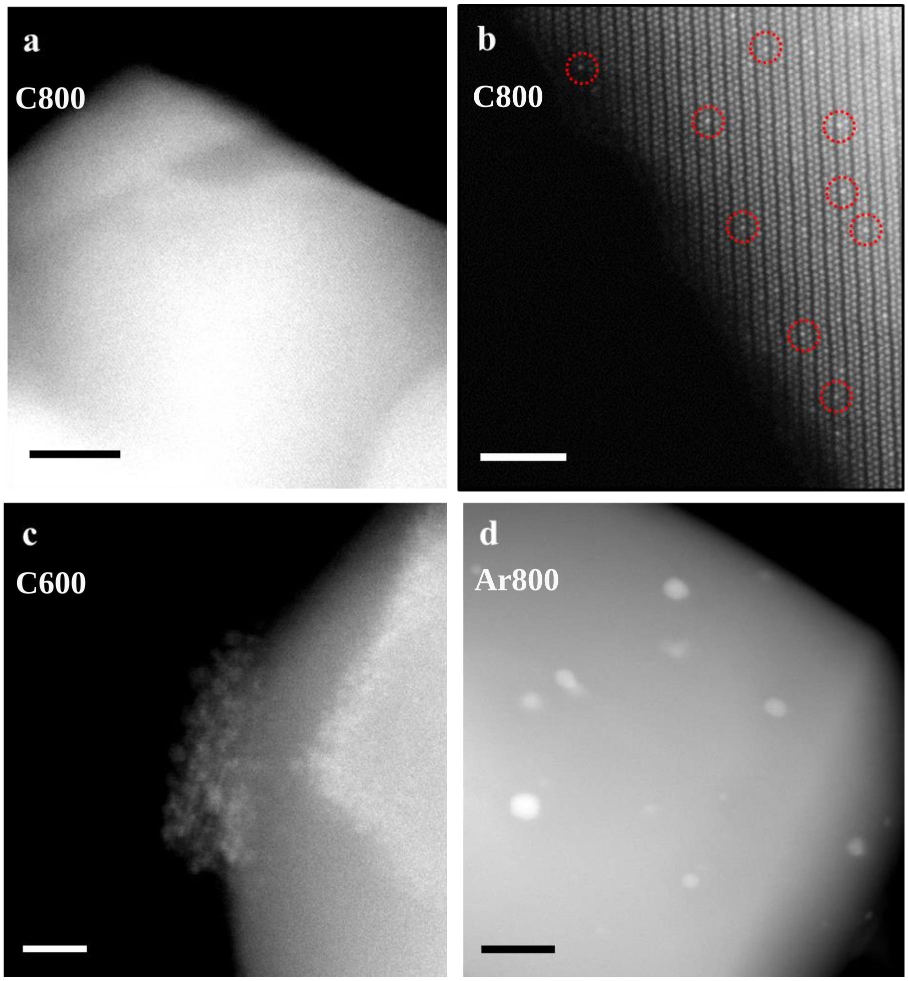

Supplementary Figure 4. Characterization of 0.3Pt/Fe_2O_3-C800, 0.3Pt/Fe_2O_3-C600 and 0.3Pt/Fe_2O_3-Ar800.

(a) Low resolution and (b) HRSTEM images of 0.3Pt/Fe_2O_3-C800 showing absence of Pt NPs and presence of isolated Pt atoms (circled in red) perfectly aligned with Fe sites in the oxide support lattice. 10 nm scale bar for panel a, and 2 nm scale bar for panel b. (c) Low resolution STEM image of 0.3Pt/Fe_2O_3-C600 resulting from a lower temperature (600 °C) calcination of the parent 0.3Pt/Fe_2O_3-NP evidences loosely coordinated Pt NPs over the support. 5 nm scale bar. (d) Low resolution STEM images of 0.3Pt/Fe_2O_3-Ar800 demonstrating Pt NP formation following 800 °C annealing under Ar. 20 nm scale bar. Thermal treatment under an oxidizing environment disperses Pt NPs, whereas treatment under an inert environment promotes their aggregation.

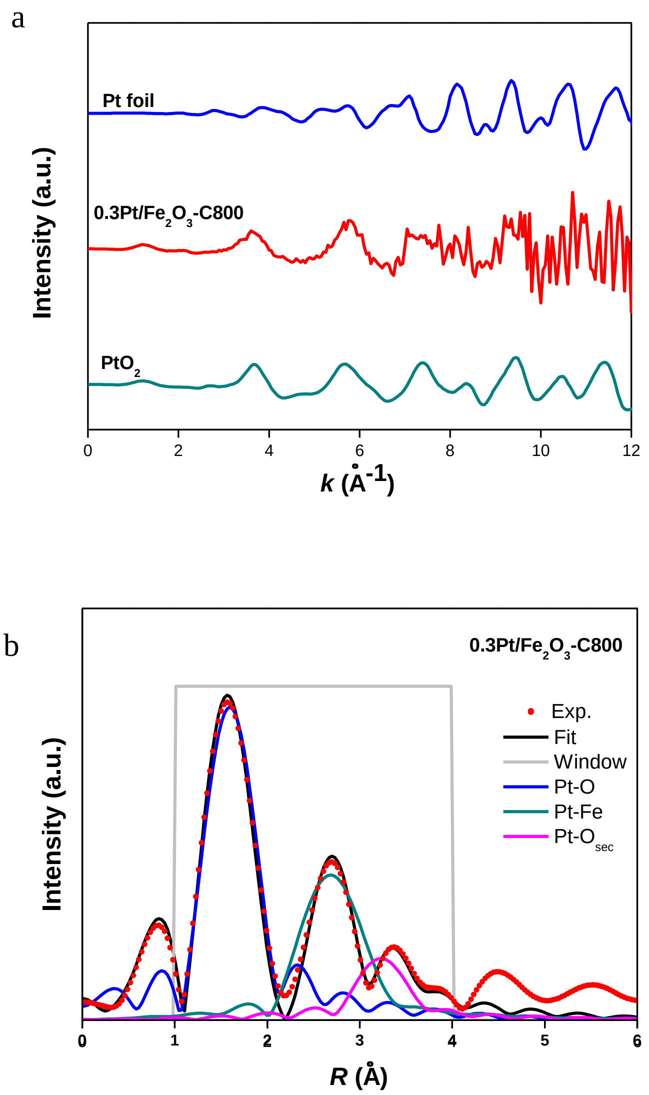

Supplementary Figure 5. EXAFS characterization.

(a) k^3-weighted EXAFS spectra of the 0.3Pt/Fe_2O_3-C800, PtO_2, and Pt foil. (b) Experimental and fitted FT radial distribution functions of 0.3Pt/Fe_2O_3-C800.

Supplementary Table 1. Fitted EXAFS parameters for Pt foil, 0.3Pt/Fe_2O_3-C800, and PtO_2.

Samples	Shell	*N*	*R* (Å)	σ^2x10^2 (Å^2)	r-factor (%)
Pt Foil	Pt-Pt	12.0	2.76	0.34	0.019
0.3Pt/Fe_2O_3-C800	Pt-O	4.20	2.00	0.04	0.009
	Pt-Fe	6.06	3.07	0.94	0.009
	Pt-O_{sec}	6.36	3.72	0.31	0.009
PtO_2	Pt-O	6.00	2.01	0.27	0.010
	Pt-Pt	6.00	3.12	0.40	0.010
	Pt-O_{sec}	12.00	3.67	0.48	0.010

N is the coordination number for the absorber-backscatterer pair. *R* is the average absorber−backscatterer distance. σ^2 is the Debye−Waller factor. The accuracies of the above parameters were estimated as *N*, ±20%; *R*, ±1%; σ^2, ±20%. The data ranges used for data fitting in *k*-space (Δk) are 3.0-9.5 Å^{-1} for 0.3Pt/Fe_2O_3-C800, and PtO_2 and 3.0-12 Å^{-1} for Pt foil; in *R*-space (ΔR) 1.0 -4.0 Å for 0.3Pt/Fe_2O_3-C800, and PtO_2, 1.0 -2.0 Å for Pt foil, respectively. Pt-$O_{sec.}$ indicates the second nearest neighbor O shell.

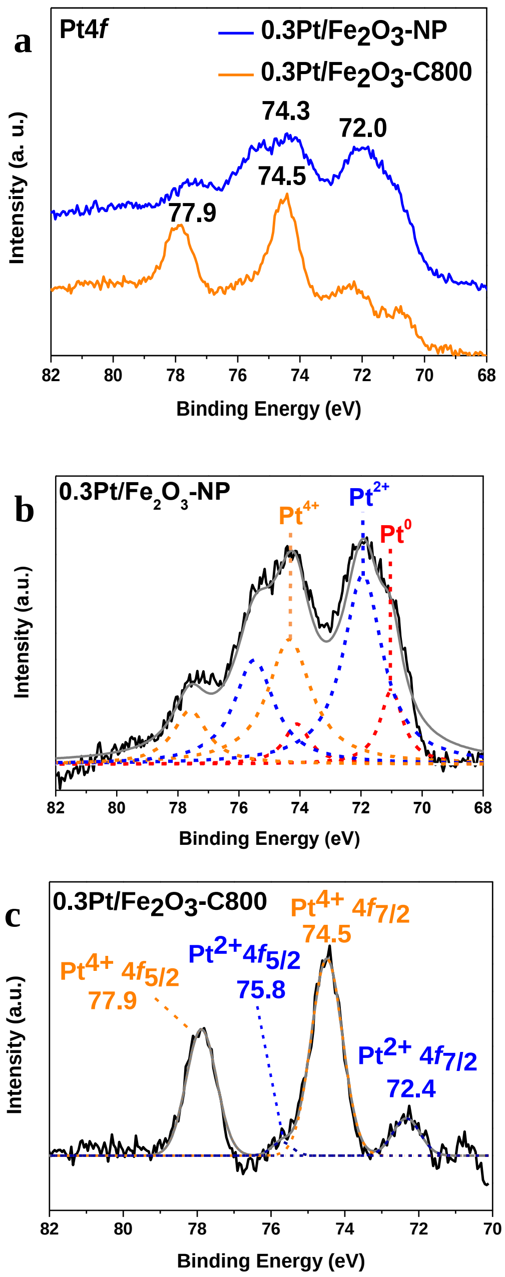

Supplementary Figure 6. Pt 4*f* XP spectra of 0.3Pt/Fe_2O_3-NP and 0.3Pt/Fe_2O_3-C800.

(a) High-resolution Pt 4*f* XP spectra of 0.3Pt/Fe_2O_3-NP and 0.3Pt/Fe_2O_3-C800. (b) Deconvolution analysis of Pt 4*f* XP spectra of 0.3Pt/Fe_2O_3-NP exhibits a mix of metallic (Pt^0 $4f_{7/2}$~71.0 eV, Pt^0 $4f_{5/2}$~74.1 eV) and partially oxidized platinum (Pt^{2+} $4f_{7/2}$~71.9 eV, Pt^{2+} $4f_{5/2}$~75.5 eV, Pt^{4+} $4f_{7/2}$~74.3 eV, Pt^{4+} $4f_{5/2}$~77.6 eV). The presence of Pt^{2+} and Pt^{4+} may come from surface oxidation of Pt NPs during calcination. (c) 0.3Pt/Fe_2O_3-C800 evidences predominantly high binding energy (+4) platinum.

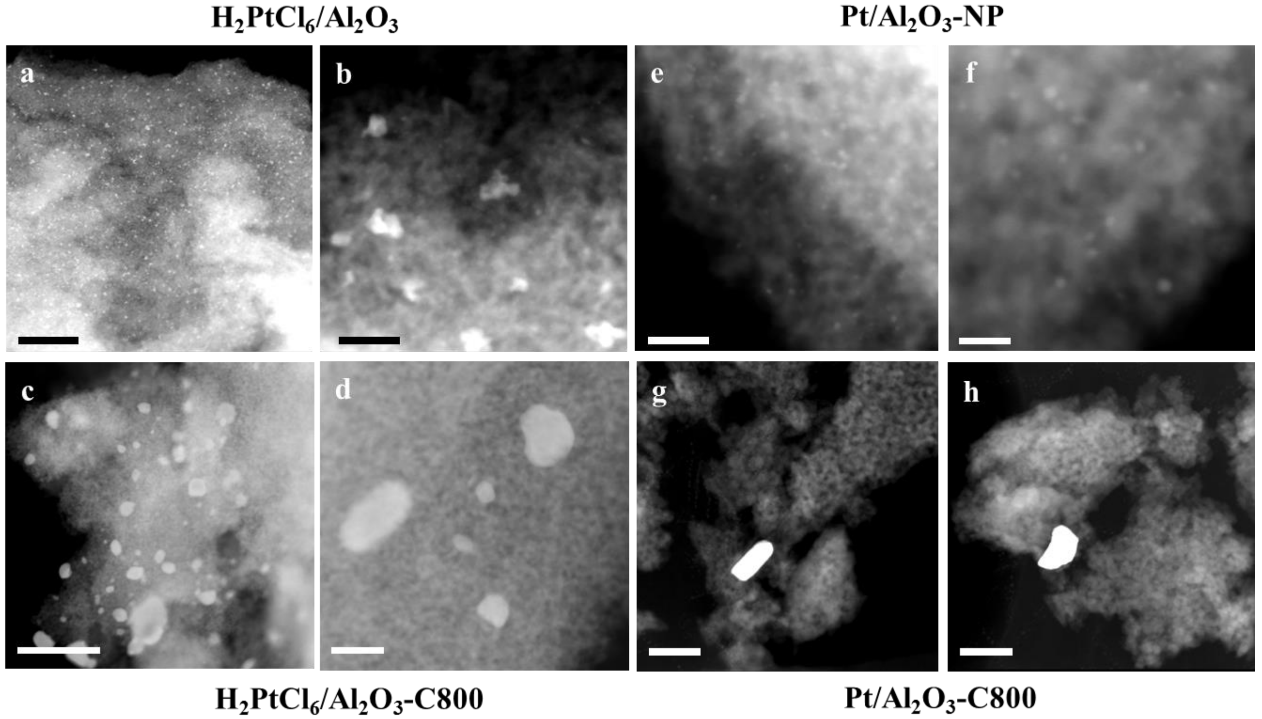

Supplementary Figure 7. Characterization of H_2PtCl_6 or Pt NP functionalized Al_2O_3 before and after calcination.

(a-b) Low resolution STEM images of 1 wt% H_2PtCl_6 deposited by aqueous wet impregnation on Al_2O_3 reveal <10 nm Pt nanoparticles. 200 nm scale bar for panel a, and 20 nm scale bar for panel b. (c-d) Subsequent high temperature calcination at 800 °C for 5 h results in significant particle sintering over alumina. 200 nm scale bar for panel c, and 50 nm scale bar for panel d. (e-f) Low resolution STEM images of 0.3 wt% Pt NP deposited on Al_2O_3 reveal 2-3 nm Pt nanoparticles. 20 nm scale bar for panel e, and 10 nm scale bar for panel f. (g-h) Calcination at 800 °C in air for 5 h results in severe aggregation, in contrast to observations over Fe_2O_3. Scale bar: 50 nm. Alumina is unable to stabilize atomically-dispersed Pt species.

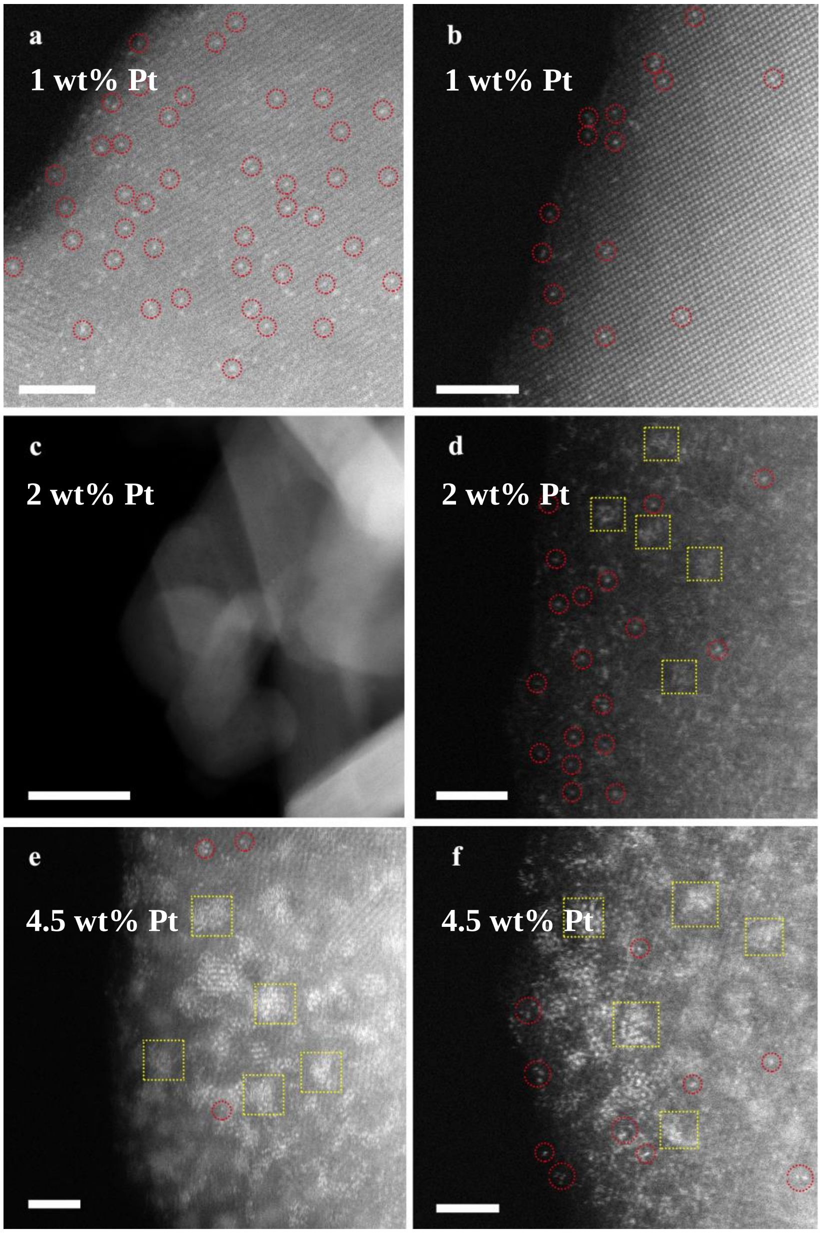

Supplementary Figure 8. Aberration-corrected HAADF-STEM images of *x*Pt/Fe_2O_3-C800 with different Pt loadings.

(a-b) HRSTEM images of 1Pt/Fe_2O_3-C800 exhibit only isolated Pt atoms (circled in red): 2 nm scale bars. (c-d) Corresponding images for 2Pt/Fe_2O_3-C800 evidence a significant number of isolated Pt atoms, accompanied by a small number of Pt clusters (highlighted in yellow squares). 50 nm scale bar for panel c, and 2 nm scale bar for panel d. (e-f) HRSTEM images of 4.5Pt/Fe_2O_3-C800 also reveal some isolated Pt atoms, accompanied by a higher density of larger Pt clusters. 2 nm scale bars.

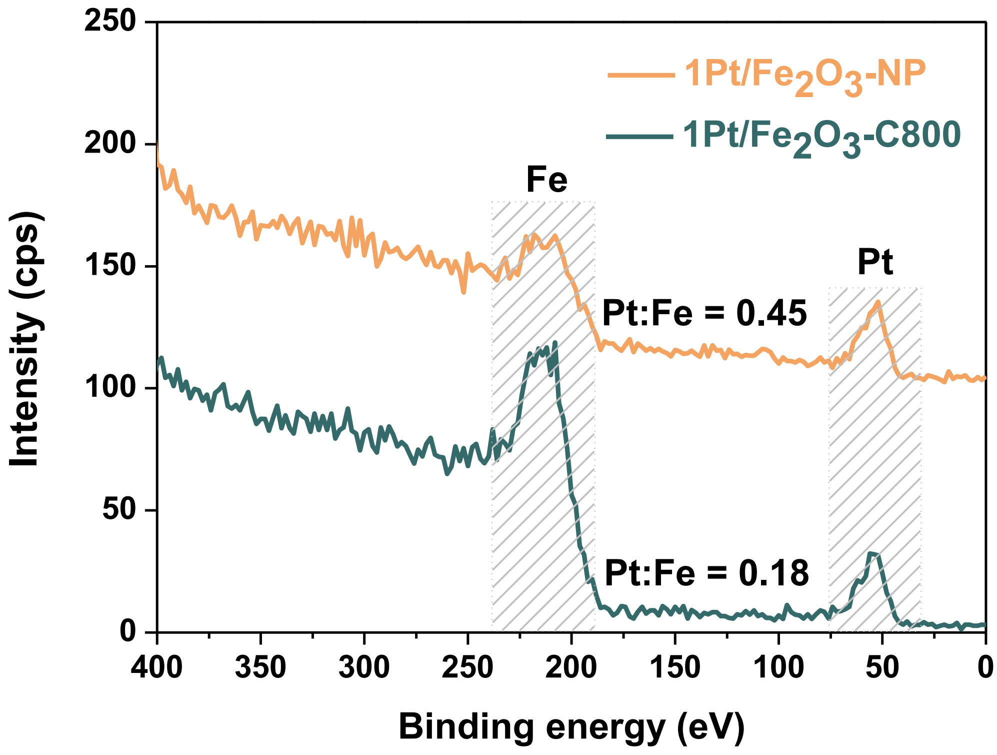

Supplementary Figure 9. LEIS data for 1Pt/Fe_2O_3-NP before and after calcination.

Low energy He^+ ion scattering spectroscopy reveals that the Pt:Fe ratio within the outermost surface layer decreased from 0.45 to 0.18 as the calcination temperature was increased from 500 °C to 800 °C under flowing air. This is consistent with migration of Pt atoms into the near sub-surface region of the Fe_2O_3 support (ICP-AES elemental analysis confirmed no loss of Pt due to evaporation).

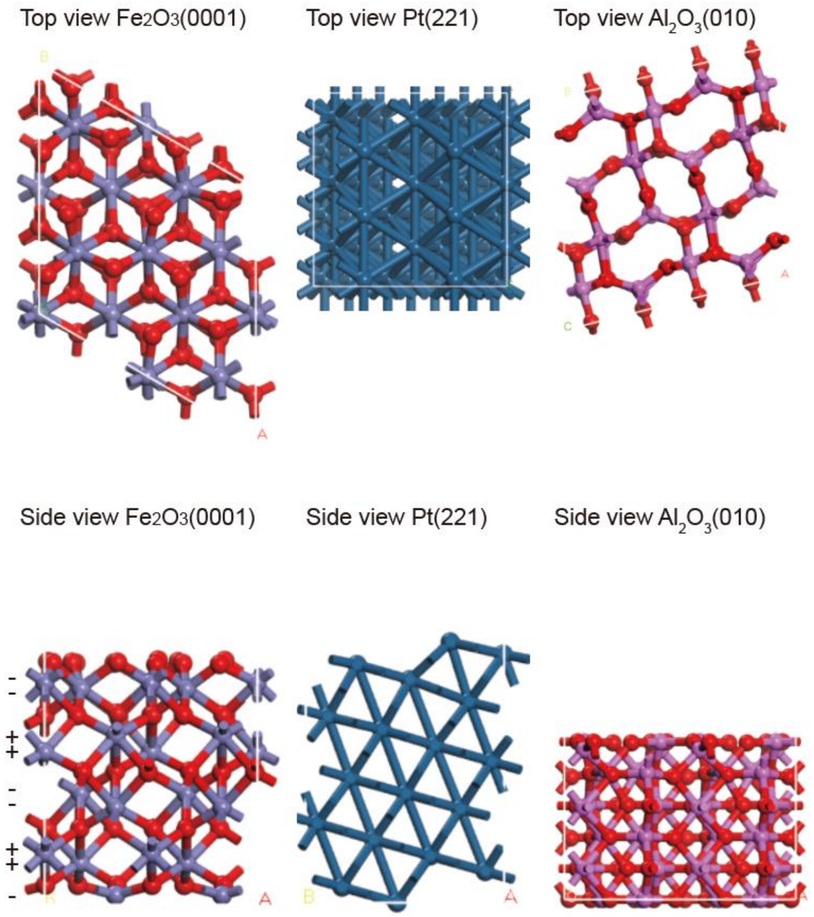

Supplementary Figure 10. Computational model of Fe_2O_3(0001), Pt(221) stepped, and θ-Al_2O_3(010) surfaces.

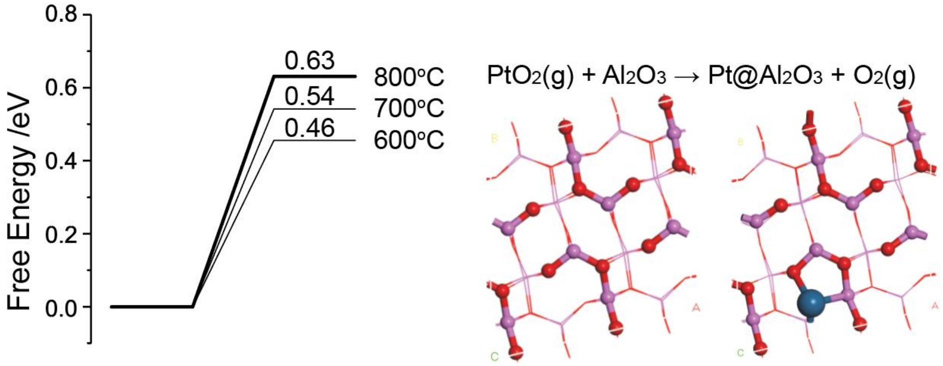

Supplementary Figure 11. **Optimized structures and free energy profiles of PtO_2 anchoring step on Al_2O_3(010) surface.**

Supplementary Table 2. Bader charge analysis of Pt atoms, and Pt-O bond lengths for gas and condensed phase PtO_2, Pt/Fe_2O_3, and Pt/Al_2O_3.

	**PtO_2(gas)**	**PtO_2(bulk)**	**Pt/Fe_2O_3**	**Pt/Al_2O_3**
**Bader charge of Pt / e^-**	+1.61	+1.73	+1.43	-0.14
**Pt-O bond length / Å**	1.70	2.04	1.90, 1.94, 1.95, 1.95	2.35, 2.36

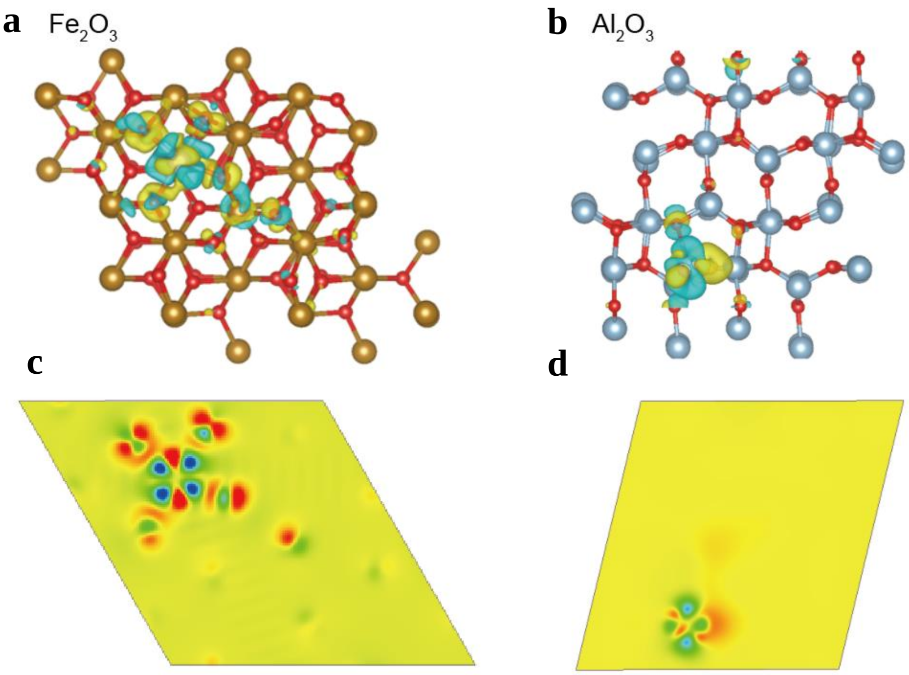

Supplementary Figure 12. Calculated charge density differences for Pt adatoms on the (a) Fe_2O_3(0001) and (b) Al_2O_3(010) surface. Yellow and blue areas represent charge increase and reduction, respectively. The cut-off of the density-difference isosurfaces equals 0.005 electrons Å^{-3}. (c, d) Two dimensional representation of charge difference at the Pt horizontal face.

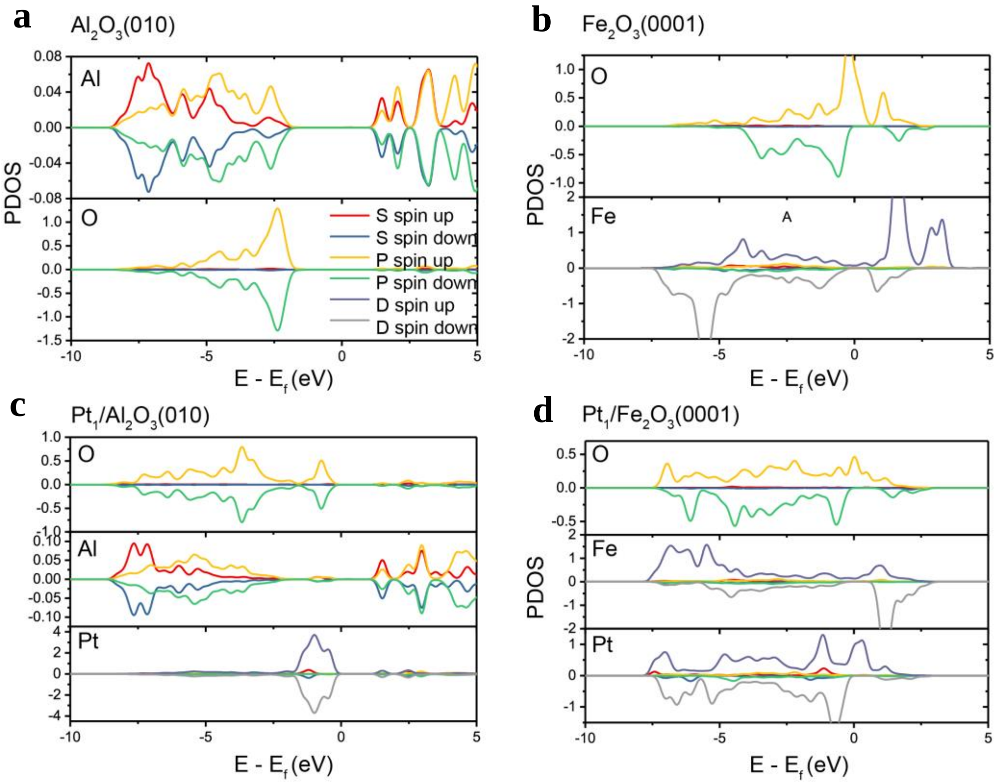

Supplementary Figure 13. Projected electronic density of states (PDOS) of O, Al, Fe, and Pt on (a) Al_2O_3(010), (b) Fe_2O_3(0001), (c) Pt_1/Al_2O_3(010), and (d) Pt_1/Fe_2O_3(0001) surfaces.

Analysis of Pt single atom stabilization over Fe_2O_3 versus Al_2O_3. The driving energy for the stabilization of Pt on Fe_2O_3 arises from Pt-O bond formation. As shown in Supplementary Figure 12, we calculated the charge density difference of Pt_1/Fe_2O_3(0001) and Pt_1/Al_2O_3(010), defined as $\Delta\rho = \rho_{Pt+slab} - \rho_{slab} - \rho_{Pt}$. For Pt_1/Fe_2O_3(0001), there are four strong chemical bonds between Pt and adjacent O resulting from *d-p* orbital interactions, which result in an oxidized Pt with +1.43 |e| from Bader charge analysis. Here +1.43 |e| is slightly lower than the formal charge of Pt in gas PtO_2 and bulk PtO_2 (Supplementary Table 2). However, the Pt-O bond lengths on the Fe_2O_3 surface are around 1.94 Å, which is even shorter than in crystalline PtO_2. In contrast, Pt-O bonds on the Al_2O_3 surface are much longer at 2.35 Å, suggesting a far weaker interaction of Pt with surface O atoms.

Comparing the projected electronic density of states (PDOS) for Fe_2O_3(0001) and Al_2O_3(010) as shown in Supplementary Figure 13, the *s* and *p* orbitals of O^{2-} at the Al_2O_3(010) surface are fully occupied, and the band gap of the Al^{3+} system is too large to accept electrons from Pt, i.e. the strong Al-O bonding network prevents any significant metal-support interaction with Pt adatoms.

This scenario contrasts with O^{n-} at the Fe_2O_3(0001) surface, where the orbitals are not fully occupied. Moreover, the reducibility of Fe^{m+} ions implies that it remains to be able to accept electrons from Pt adatoms, thus favoring a strong metal-support interaction.

Details of free energy calculation. The chemical equation of evaporation of one row of Pt or one row of PtO_2, i.e. three Pt or three PtO_2 in each slab model, at step of Pt(221) can be written as:

(1) Pt(221 slab) → Pt(221 slab, with one row Pt evaporated) + $3Pt_1$(gas)
(2) Pt(oxygen covered 221 slab) → Pt(oxygen covered 221 slab, with one row PtO_2 evaporated) + $3PtO_2$(gas)

The chemical equation for adding PtO_2 to Fe_2O_3(0001) or θ-Al_2O_3(010) surfaces is:

(3) PtO_2(g) + Fe_2O_3 → Pt_1@Fe_2O_3 + O_2(g)
(4) PtO_2(g) + Al_2O_3 → Pt@Al_2O_3 + O_2(g)

Thus, the energy changes at 0 K (neglecting zero point energy (ZPE)) are:

$\Delta E(1) = (E_{\text{Pt(221 slab, with one row Pt evaporated)}} + 3E_{\text{Pt(gas)}} - E_{\text{Pt(221 slab)}})/3 = 6.00$ eV

$\Delta E(2) = (E_{\text{Pt(oxygen covered 221 slab, with one row PtO2 evaporated)}} + 3E_{\text{PtO2(gas)}} - E_{\text{Pt(oxygen covered 221 slab)}})/3 = 2.38$ eV

$\Delta E(3) = E_{\text{Pt1@Fe2O3}} + E_{\text{O2(g)}} - E_{\text{Fe2O3}} + E_{\text{PtO2(g)}} = -3.16$ eV

$\Delta E(4) = E_{\text{Pt1@Al2O3}} + E_{\text{O2(g)}} - E_{\text{Al2O3}} + E_{\text{PtO2(g)}} = -0.07$ eV

The standard Gibbs free energies of $G_{\text{O2(g)}}$, $G_{\text{Pt(g)}}$, and $G_{\text{PtO2(g)}}$ were calculated using the following equations, taking into account the individual translational E_t and S_t, vibrational E_v and S_v, rotational E_r and S_r, and ZPE contributions:

$$G = H - TS = U + k_bT - TS$$

$$S = S_t + S_v + S_r$$

$$U = E_{\text{DFT}} + \text{ZPE} + E_t + E_v + E_r$$

where E_{DFT} are the electronic energies from DFT calculations. S_t, S_v, S_r, E_t, E_v, and E_r are obtained by including partition functions, Q, according to:

$$U = k_bT^2\left(\frac{\partial \ln Q}{\partial T}\right)_{N,V}$$

$$S = k_b \ln Q + k_b T (\frac{\partial \ln Q}{\partial T})_{N,V}$$

$$\ln Q = N \left[\ln \left(\frac{q_{trans}}{N} \right) + 1 \right] + N \ln q_{rot} + N \ln q_{vib} + N \ln q_{ele}$$

For slab models, the entropy and enthalpy corrections to free energies are neglected in this work. The resulting corrections to the ZPE, *H*, *S*, and *G* at various temperatures are given below:

Supplementary Table 3. Free energy corrections of Pt(g), PtO_2(g), and O_2(g) at 873, 973, and 1073 K.

		ZPE (eV)	$H_{0\rightarrow T}$ (eV)	S (eV/K)	$G_{0\rightarrow T}$ (eV)
Pt(gas)	873 K	0.00	0.19	0.00204	-1.59
	973 K	0.00	0.21	0.00206	-1.80
	1073 K	0.00	0.23	0.00208	-2.01
PtO_2(gas)	873 K	0.14	0.60	0.00333	-2.31
	973 K	0.14	0.66	0.00340	-2.65
	1073 K	0.14	0.72	0.00346	-2.99
O_2(gas)	873 K	0.10	0.38	0.00248	-1.79
	973 K	0.10	0.41	0.00252	-2.04
	1073 K	0.10	0.45	0.00256	-2.29

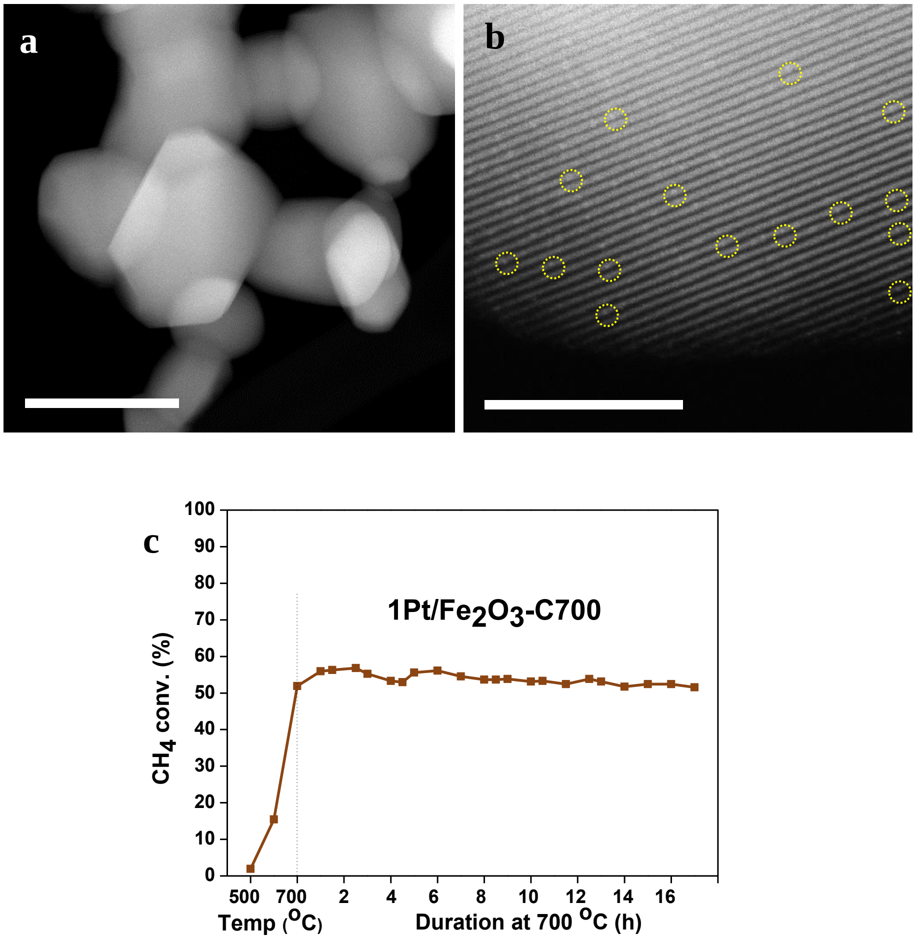

Supplementary Figure 14. Aberration-corrected HAADF-STEM images of the used 1Pt/Fe_2O_3-NP catalyst, and catalytic performance of Pt SAC.

(a) STEM showed no evidence of Pt NP in the 1Pt/Fe_2O_3-NP catalyst following 6 h methane oxidation at 700 °C, rather (b) HRSTEM evidenced only isolated Pt atoms (circled in yellow), demonstrating that Pt NPs are dispersed into single atoms during reaction. 200 nm scale bar for panel a, and 5 nm scale bar for panel b. (c) 1Pt/Fe_2O_3-NP was pre-treated under 10 vol% O_2/He flow (30 mL min^{-1}) at 700 °C for 5 h to form a Pt SAC (1Pt/Fe_2O_3-C700). The gas feed was subsequently switched to 0.5 vol% CH_4 / 3 vol% O_2 / 97 % He balance (30 mL min^{-1}) to evaluate its performance in methane oxidation. CH_4 conversion remained stable ~50 % over the Pt SAC for 16 h at 700 °C despite the harsh reaction conditions.

Supplementary Table 4. Reactivity comparation of 1Pt/Fe_2O_3-NP and 1Pt/Fe_2O_3-C700.

Entry	Usage (mg)	Space velocity ($mL\ h^{-1}\ g_{cat}^{-1}$)	Specific rate ($mol_{CH4}\ h^{-1}\ g_{Pt}^{-1}$)	TOF (s^{-1})
1Pt/Fe_2O_3-NP[a]	17	176,000	0.47	0.086
1Pt/Fe_2O_3-C700[a]	6	500,000	2.01	0.1
Pt/Al_2O_3[b]	200		0.093	

[a]The gas feed was 0.5 vol% CH_4 / 3 vol% O_2 / 97 % He balance (50 mL min^{-1}) to control the CH_4 conversion at 700 °C below 20 %. [b]Estimated from ref [1]: 100 % CH_4 conversion, 1.9 wt% Pt.

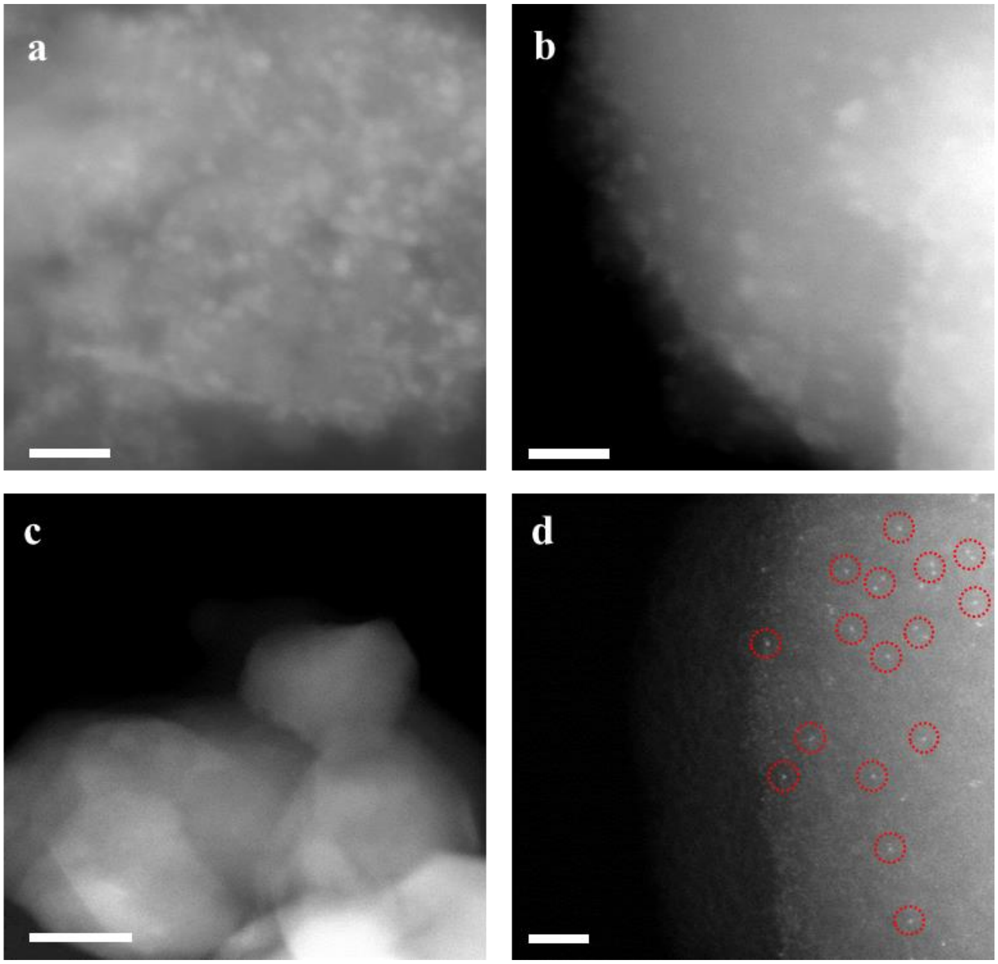

Supplementary Figure 15. STEM images of H_2PtCl_6/Fe_2O_3 before and after calcination.
(a-b) HRSTEM images of 1 wt% H_2PtCl_6 deposited on Fe_2O_3 and heated to 300 °C for 5 h under air showing the formation of small Pt NPs. 10 nm scale bar for panel a, and 5 nm scale bar for panel b. (c) Low-resolution STEM image, and (d) AC-HRSTEM images following 800 °C calcination under air for 5 h, demonstrate complete dispersion of Pt NPs as isolated atoms (circled in red). 20 nm scale bar for panel c, and 2 nm scale bar for panel d.

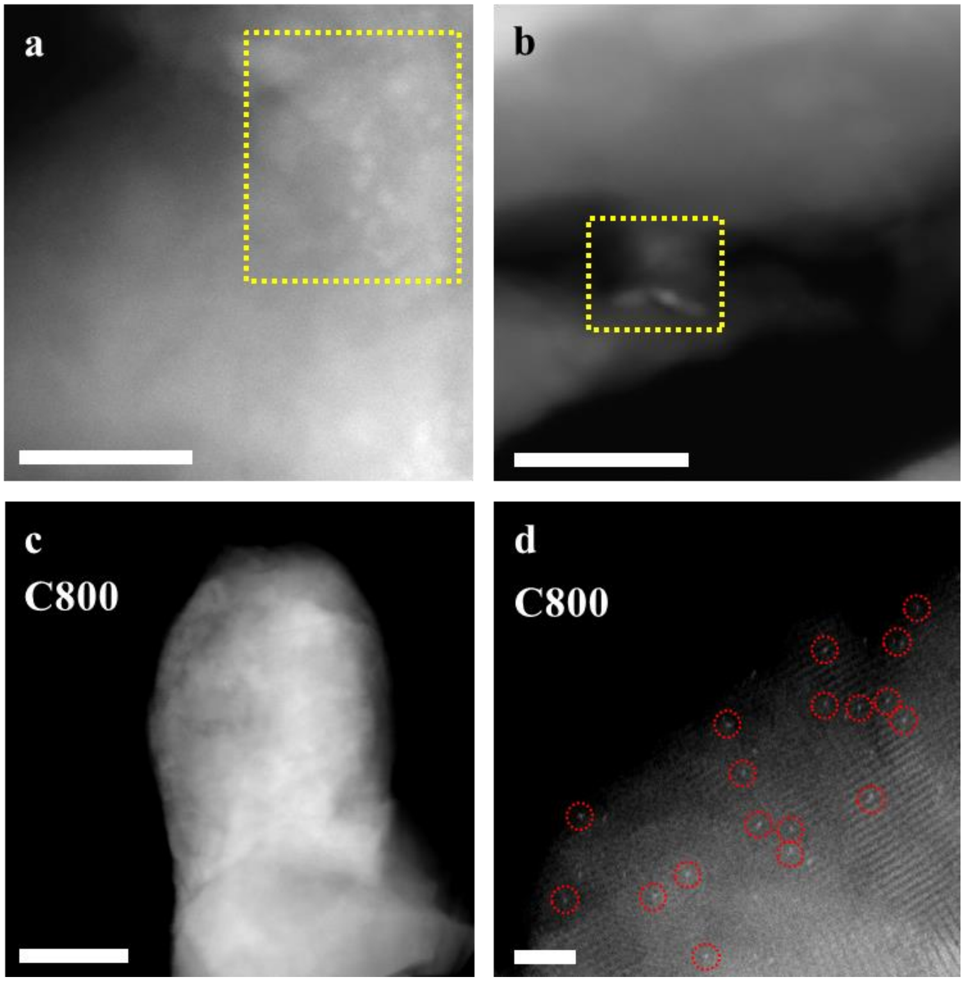

Supplementary Figure 16. STEM images of H_2PtCl_6/Fe_2O_3(N) before and after calcination. (a-b) HRSTEM images of 0.2 wt% H_2PtCl_6 deposited on Fe_2O_3(N) and heated to 300 °C for 5 h under air showing the formation of small Pt NPs (highlighted in yellow squares). 20 nm scale bars. (c) Low-resolution STEM image, and (d) AC-HRSTEM images following 800 °C calcination under air for 5 h, demonstrate complete dispersion of Pt NPs as isolated atoms (circled in red). 50 nm scale bar for panel c, and 2 nm scale bar for panel d.

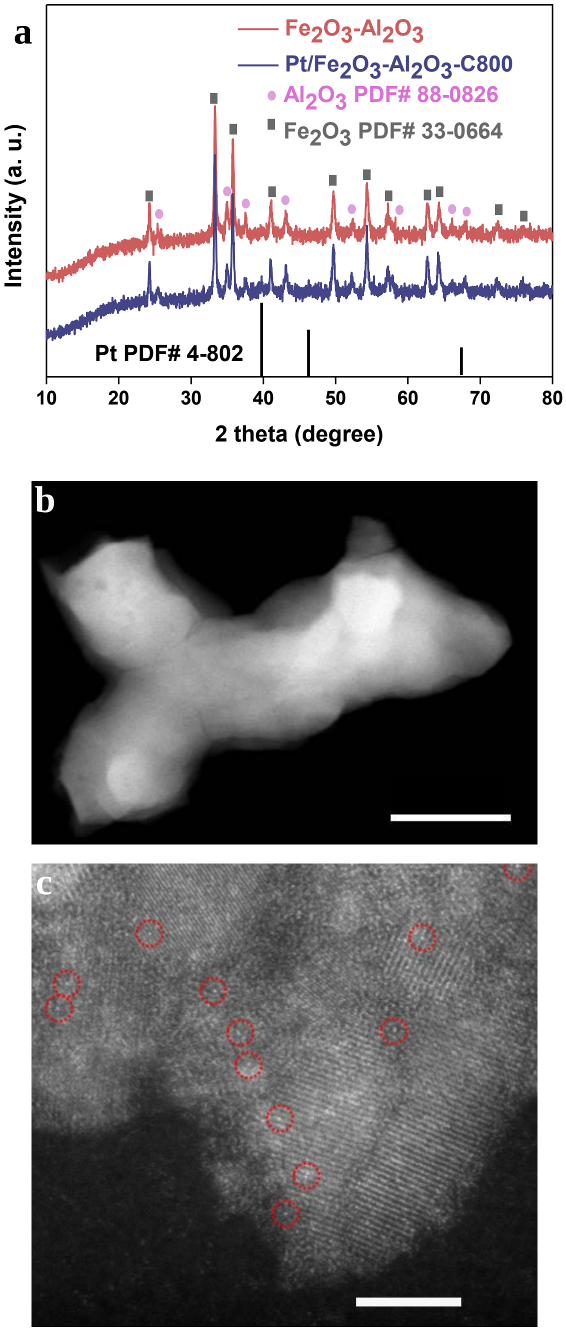

Supplementary Figure 17. Characterization of Pt/Fe_2O_3-Al_2O_3-C800.

(a) XRD pattern of Fe_2O_3-Al_2O_3 support and Pt/Fe_2O_3-Al_2O_3-C800 evidence only reflections characteristic of Al_2O_3 and Fe_2O_3, but none of any Pt phase. (b) Low resolution, and (c) HRSTEM images confirm the absence of any Pt NP over the Fe_2O_3-Al_2O_3 support and existence of atomically-dispersed Pt atoms (circled in red). 100 nm scale bar for panel b, and 5 nm scale bar for panel c.

Supplementary Appendix I: Experimental studies of single atoms located at support defects.

Entry	sample	location	ref
1	Pd/MgO	O vacancy	2
2	Pt/Al_2O_3	Al^{3+}_{penta}	3
3	Pd/Al_2O_3	defect	4
4	Fe/SiO_2	Si vacancy	5
5	Pt/C	defect	6
6	Pd/C	defect	7
7	Pt/C	vacancy	8
8	Fe, Co, Ni/C	vacancy	9
9	Pd/C	vacancy	10
10	Co-Pt/C	defect	11
11	$Pd/mpg\text{-}C_3N_4$	"six-fold Cavities"	12
12	Ir,Au,Pd,Ag,Pt/C	defect	13
13	Pd/graphene	vacancies	14
14	Pt/graphene	defects	15
15	Pt,Co, In/graphene	vacancies	16
16	Pt/MoS_2	S vacancy	17
17	Co/MoS_2	defect	18
18	Pt/CeO_2	defects	19
19	Au/CeO_2	O defect	20
20	Au/CeO_2	O vacancy	21
21	Au/CeO_2	Ce vacancy	22
22	Pt/CeO_2	step edges	23
23	Pt/CeO_2	Nano pocket	24
24	Pt/CeO_2	O vacancy	25
25	Pt/FeO_x	O vacancy	26
26	$Co/Fe_3O_4(001)$	octahedral vacancies	27
27	$Ni/Fe_3O_4(001)$	cation vacancies	28
28	Cu, $Ag/Fe_3O_4(001)$	"narrow" site	29
29	$Au/Fe_3O_4(001)$	"narrow" hollow site	30
30	Ni, Co, Mn, Ti, and Zr/Fe_3O_4	cation vacancy	31
31	Rh/CoO	O vacancy	32.
32	$Pt/Ni(OH)_x$	Ni^{2+} vacancy	33
33	Pt, Au/ZnO	Zn vacancy	34
34	Rh/ZnO	vacancy	35
35	Au/TiO_2	O vacancy	36
36	Pt/TiO_2	Defect	37
37	Pt/TiO_2	O vacancy	38
38	Pd/TiO_2	defects	39
39	Pt/WO_x	O vacancy	40

Supplementary Appendix II: DFT studies of single atoms located at support defects.

Entry	sample	location	ref
1	Pt/NB	defects	[41]
2	Pd/NB	B vacancy	[42]
3	Fe/MoS_2	S vacancy	[43]
4	Rh/CoO	O vacancy	[44]
5	Co/graphene	vacancy	[45]
6	Au/graphene	defect	[46]
7	M/FeO_x (M = Au, Rh, Pd, Co, Cu, Ru and Ti)	O vacancy	[47]
8	Au/CeO_2	O vacancy	[48]
9	Au/CeO_2	O defect	[49]

Supplementary Appendix III: Reviews citing the localization of single atoms at support defects.

Entry	Title	ref
1	Preparation, characterization and catalytic performance of single-atom catalysts.	[50]
2	Increasing the range of non-noble-metal single-atom catalysts.	[51]
3	Two-dimensional materials confining single atoms for catalysis.	[52]
4	Atomically dispersed supported metal catalysts.	[53]
5	Single-atom catalysts: A new frontier in heterogeneous catalysis.	[54]
6	Catalysis by supported single metal atoms.	[55]
7	The power of single-atom catalysis.	[56]
8	Atomically dispersed supported metal catalysts: perspectives and suggestions for future research.	[57]
9	Single-atom electrocatalysts.	[58]
10	Metal catalysts for heterogeneous catalysis: from single atoms to nanoclusters and nanoparticles.	[59]
11	Strategies for stabilizing atomically dispersed metal catalysts.	[60]
12	Single-atom catalysts: emerging multifunctional materials in heterogeneous catalysis	[61]
13	Heterogeneous single-atom catalysis.	[62]
14	Unravelling single atom catalysis: The surface science approach.	[63]

Supplementary References

1 Urfels, L., Gélin, P., Primet, M. & Tena, E. Complete oxidation of methane at low temperature over Pt catalysts supported on high surface area SnO_2. *Top. Catal.* **30**, 427-432 (2004).

2 Abbet, S. *et al.* Acetylene cyclotrimerization on supported size-selected Pd_n clusters ($1 \leqslant n \leqslant 30$): one atom is enough! *J. Am. Chem. Soc.* **122**, 3453-3457 (2000).

3 Kwak, J. H. *et al.* Coordinatively unsaturated Al^{3+} centers as binding sites for active catalyst phases of platinum on γ-Al_2O_3. *Science* **325**, 1670-1673 (2009).

4 Hackett, S. F. J. *et al.* High-activity, single-site mesoporous Pd/Al_2O_3 catalysts for selective aerobic oxidation of allylic alcohols. *Angew. Chem., Int. Ed.* **46**, 8593-8596 (2007).

5 Hu, B. *et al.* Isolated Fe^{II} on silica as a selective propane dehydrogenation catalyst. *ACS Catal.* **5**, 3494-3503 (2015).

6 Wei, H. *et al.* Iced photochemical reduction to synthesize atomically dispersed metals by suppressing nanocrystal growth. *Nat. Commun.* **8**, 1490 (2017).

7 Wei, S. *et al.* Direct observation of noble metal nanoparticles transforming to thermally stable single atoms. *Nat. Nanotechnol.* **13**, 856-861 (2018).

8 Bulushev, D. A. *et al.* Single atoms of Pt-group metals stabilized by N-doped carbon nanofibers for efficient hydrogen production from formic acid. *ACS Catal.* **6**, 3442-3451 (2016).

9 Fei, H. *et al.* General synthesis and definitive structural identification of MN_4C_4 single-atom catalysts with tunable electrocatalytic activities. *Nat. Catal.* **1**, 63-72 (2018).

10 Bulushev, D. A. *et al.* Single isolated Pd^{2+} cations supported on N-doped carbon as active sites for hydrogen production from formic acid decomposition. *ACS Catal.* **6**, 681-691 (2016).

11 Zhang, L. *et al.* Coordination of atomic Co-Pt coupling species at carbon defects as active sites for oxygen reduction reaction. *J. Am. Chem. Soc.* **140**, 10757-10763 (2018).

12 Vile, G. *et al.* A stable single-site palladium catalyst for hydrogenations. *Angew. Chem. Int. Ed.* **54**, 11265-11269 (2015).

13 Chen, Z. *et al.* Stabilization of single metal atoms on graphitic carbon nitride. *Adv. Funct. Mater.* **27**, 1605785 (2017).

14 Yan, H. *et al.* Single-atom Pd_1/graphene catalyst achieved by atomic layer deposition: remarkable performance in selective hydrogenation of 1,3-butadiene. *J. Am. Chem. Soc.* **137**, 10484-10487 (2015).

15 Sun, S. *et al.* Single-atom catalysis using Pt/graphene achieved through atomic layer deposition. *Sci. Rep.* **3**, 1775 (2013).

16 Wang, H. *et al.* Doping monolayer graphene with single atom substitutions. *Nano Lett.* **12**, 141-144 (2012).

17 Li, H. *et al.* Atomic structure and dynamics of single platinum atom interactions with monolayer MoS_2. *ACS Nano* **11**, 3392-3403 (2017).

18 Liu, G. *et al.* MoS_2 monolayer catalyst doped with isolated Co atoms for the hydrodeoxygenation reaction. *Nat. Chem.* **9**, 810-816 (2017).

19 Jones, J. *et al.* Thermally stable single-atom platinum-on-ceria catalysts via atom trapping. *Science* **353**, 150-154 (2016).

20 Fu, Q., Deng, W., Saltsburg, H. & Flytzani-Stephanopoulos, M. Activity and stability of low-content gold–cerium oxide catalysts for the water–gas shift reaction. *Appl. Catal., B* **56**, 57-68 (2005).
21 Fu, Q., Saltsburg, H. & Flytzani-Stephanopoulos, M. Active nonmetallic Au and Pt species on ceria-based water-gas shift catalysts. *Science* **301**, 935-938 (2003).
22 Qiao, B. *et al.* Highly efficient catalysis of preferential oxidation of CO in H_2-rich stream by gold single-atom catalysts. *ACS Catal.* **5**, 6249-6254 (2015).
23 Dvorak, F. *et al.* Creating single-atom Pt-ceria catalysts by surface step decoration. *Nat. Commun.* **7**, 10801 (2016).
24 Bruix, A. *et al.* Maximum noble-metal efficiency in catalytic materials: atomically dispersed surface platinum. *Angew. Chem. Int. Ed.* **53**, 10525-10530 (2014).
25 Xie, P. *et al.* Nanoceria-supported single-atom platinum catalysts for direct methane conversion. *ACS Catal.*, 4044-4048 (2018).
26 Wei, H. *et al.* FeO_x-supported platinum single-atom and pseudo-single-atom catalysts for chemoselective hydrogenation of functionalized nitroarenes. *Nat. Commun.* **5**, 5634 (2014).
27 Gargallo-Caballero, R. *et al.* Co on Fe_3O_4(001): Towards precise control of surface properties. *J. Chem. Phys.* **144**, 094704 (2016).
28 Ryan, P. T. P. *et al.* Direct measurement of Ni incorporation into Fe_3O_4(001). *Phys Chem Chem Phys* **20**, 16469-16476 (2018).
29 Meier, M. *et al.* Probing the geometry of copper and silver adatoms on magnetite: quantitative experiment versus theory. *Nanoscale* **10**, 2226-2230 (2018).
30 Novotny, Z. *et al.* Ordered array of single adatoms with remarkable thermal stability: Au/Fe_3O_4(001). *Phys. Rev. Lett.* **108**, 216103 (2012).
31 Bliem, R. *et al.* Adsorption and incorporation of transition metals at the magnetite Fe_3O_4(001) surface. *Phys. Rev. B* **92**, 075440 (2015).
32 Zhang, S. *et al.* Catalysis on singly dispersed bimetallic sites. *Nat. Commun.* **6**, 7938 (2015).
33 Zhang, J. *et al.* Cation vacancy stabilization of single-atomic-site $Pt_1/Ni(OH)_x$ catalyst for diboration of alkynes and alkenes. *Nat. Commun.* **9**, 1002 (2018).
34 Gu, X.-K. *et al.* Supported single Pt_1/Au_1 atoms for methanol steam reforming. *ACS Catal.* **4**, 3886-3890 (2014).
35 Lang, R. *et al.* Hydroformylation of olefins by a rhodium single-atom catalyst with activity comparable to $RhCl(PPh_3)_3$. *Angew. Chem. Int. Ed.* **55**, 16054-16058 (2016).
36 Wan, J. *et al.* Defect effects on TiO_2 nanosheets: stabilizing single atomic site Au and promoting catalytic properties. *Adv. Mater.* **30**, doi: 10.1002/adma.201705369 (2018).
37 DeRita, L. *et al.* Catalyst architecture for stable single atom dispersion enables site-specific spectroscopic and reactivity measurements of CO adsorbed to Pt atoms, oxidized Pt clusters, and metallic Pt clusters on TiO_2. *J. Am. Chem. Soc.* **139**, 14150-14165 (2017).
38 Sasahara, A., Pang, C. L. & Onishi, H. Probe microscope observation of platinum atoms deposited on the TiO_2(110)-(1 × 1) surface. *J. Phys. Chem. B* **110**, 13453-13457 (2006).
39 Liu, P. *et al.* Photochemical route for synthesizing atomically dispersed palladium catalysts. *Science* **352**, 797-800 (2016).
40 Wang, J. *et al.* Hydrogenolysis of glycerol to 1,3-propanediol under low hydrogen pressure over WO_x -supported single/pseudo-single atom Pt catalyst. *ChemSusChem* **9**, 784-790 (2016).

41 Liu, X., Duan, T., Meng, C. & Han, Y. Pt atoms stabilized on hexagonal boron nitride as efficient single-atom catalysts for CO oxidation: a first-principles investigation. *RSC Adv.* **5**, 10452-10459 (2015).
42 Lu, Z. *et al.* Pd_1/BN as a promising single atom catalyst of CO oxidation: a dispersion-corrected density functional theory study. *RSC Adv.* **5**, 84381-84388 (2015).
43 Ma, D. *et al.* CO catalytic oxidation on iron-embedded monolayer MoS_2. *Appl. Surf. Sci.* **328**, 71-77 (2015).
44 Ma, X. L., Liu, J. C., Xiao, H. & Li, J. Surface single-cluster catalyst for N_2-to-NH_3 thermal conversion. *J. Am. Chem. Soc.* **140**, 46-49 (2018).
45 Tang, Y. *et al.* Adsorption behavior of Co anchored on graphene sheets toward NO, SO_2, NH_3, CO and HCN molecules. *Appl. Surf. Sci.* **342**, 191-199 (2015).
46 Liu, X. *et al.* Defect stabilized gold atoms on graphene as potential catalysts for ethylene epoxidation: a first-principles investigation. *Catal. Sci. Technol.* **6**, 1632-1641 (2016).
47 Li, F., Li, Y., Zeng, X. C. & Chen, Z. Exploration of high-performance single-atom catalysts on support M_1/FeO_x for CO oxidation via computational study. *ACS Catal.* **5**, 544-552 (2015).
48 Camellone, M. F. & Fabris, S. Reaction mechanisms for the CO oxidation on Au/CeO_2 catalysts: activity of substitutional Au^{3+}/Au^+ cations and deactivation of supported Au^+ adatoms. *J. Am. Chem. Soc.* **131**, 10473-10483 (2009).
49 Wang, Y.-G., Mei, D., Glezakou, V.-A., Li, J. & Rousseau, R. Dynamic formation of single-atom catalytic active sites on ceria-supported gold nanoparticles. *Nat. Commun.* **6**, 6511 (2015).
50 Wang, L. Q. *et al.* Preparation, characterization and catalytic performance of single-atom catalysts. *Chin. J. Catal.* **38**, 1528-1539 (2017).
51 Deng, T., Zheng, W. T. & Zhang, W. Increasing the range of non-noble-metal single-atom catalysts. *Chin. J. Catal.* **38**, 1489-1497 (2017).
52 Wang, Y., Zhang, W. H., Deng, D. H. & Bao, X. H. Two-dimensional materials confining single atoms for catalysis. *Chin. J. Catal.* **38**, 1443-1453 (2017).
53 Flytzani-Stephanopoulos, M. & Gates, B. C. Atomically dispersed supported metal catalysts. *Annu. Rev. Chem. Biomol. Eng.* **3**, 545-574 (2012).
54 Yang, X.-F. *et al.* Single-atom catalysts: a new frontier in heterogeneous catalysis. *Acc. Chem. Res.* **46**, 1740-1748 (2013).
55 Liu, J. Catalysis by supported single metal atoms. *ACS Catal.* **7**, 34-59 (2016).
56 Liang, S., Hao, C. & Shi, Y. The power of single-atom catalysis. *ChemCatChem* **7**, 2559-2567 (2015).
57 Gates, B. C., Flytzani-Stephanopoulos, M., Dixon, D. A. & Katz, A. Atomically dispersed supported metal catalysts: perspectives and suggestions for future research. *Catal. Sci. Technol.* **7**, 4259-4275 (2017).
58 Zhu, C., Fu, S., Shi, Q., Du, D. & Lin, Y. Single-atom electrocatalysts. *Angew. Chem. Int. Ed.* **56**, 13944-13960 (2017).
59 Liu, L. & Corma, A. Metal catalysts for heterogeneous catalysis: from single atoms to nanoclusters and nanoparticles. *Chem. Rev.* **118**, 4981-5079 (2018).
60 Qin, R., Liu, P., Fu, G. & Zheng, N. Strategies for stabilizing atomically dispersed metal catalysts. *Small Methods* **2**, 1700286 (2018).
61 Zhang, H., Liu, G., Shi, L. & Ye, J. Single-atom catalysts: emerging multifunctional materials in heterogeneous catalysis. *Adv. Energy Mater.* **8**, 1701343 (2018).

62 Wang, A. Q., Li, J. & Zhang, T. Heterogeneous single-atom catalysis. *Nat. Rev. Chem.* **2**, 65-81 (2018).
63 Parkinson, G. S. Unravelling single atom catalysis:The surface science approach. *Chin. J. Catal.* **38**, 1454-1459 (2017).
