# Supplementary material for: Non defect-stabilized thermally stable single-atom catalyst
Source: Nat Commun. 2019 Jan 16;10:234. doi: 10.1038/s41467-018-08136-3 (PMC6335577; doi:10.1038/s41467-018-08136-3)
Supplement: Supplementary file 3 — Description of Additional Supplementary Files [file 41467_2018_8136_MOESM3_ESM.pdf]

Description of Additional Supplementary Files

File Name: Supplementary Movie 1
Description: In situ TEM characterization of Pt nanoparticle oxidative dispersion.
